# Coexistence of a Leaky SCID Phenotype With Hyperphenylalaninemia in an Adult Case

**DOI:** 10.1155/crii/9988821

**Published:** 2025-03-19

**Authors:** Ugur Musabak, Tuba Erdogan, Muserref Sule Akcay, Serdar Ceylaner

**Affiliations:** ^1^Division of Immunology and Allergy, Department of Internal Medicine, Faculty of Medicine, Baskent University, Ankara, Türkiye; ^2^Department of Pulmonary Disease, Faculty of Medicine, Baskent University, Ankara, Türkiye; ^3^Department of Medical Genetics, Lokman Hekim University, Ankara, Türkiye

**Keywords:** Artemis gene, hyperphenylalaninemia, phenylketonuria, severe combined immunodeficiency

## Abstract

In recent years, due to the widespread use of advanced molecular diagnostic methods, it has become clear that individuals in particular born from consanguineous marriages may be carriers of different genetic diseases. For this reason, cases where diseases related to inborn errors of immunity (IEI) and metabolism errors are detected in the same patient are encountered more frequently. In patients affected by different genetic defects, the pathophysiology is more complex, and disease management is more difficult. In this article, we aimed to draw attention to this complex genetic carrier state in a male with primary immunodeficiency (PID). In the patient who presented with recurrent lower respiratory tract infections, bronchiectasis, asthma and nasal polyps, and antibody deficiencies as well as cellular immunodeficiency findings were detected in the immunological analyses. In the whole exome sequencing (WES) study, three different variants were detected, two in genes related to PIDs (DCLRE1C and TNFRSF13B) and one in the gene related to phenylalanine metabolism (phenylalanine hydroxylase (PAH)). In the light of the current findings, the patient was evaluated as having leaky severe combined immunodeficiency (SCID) with immune phenotype T−B−natural killer (NK)+ and hyperphenylalaninemia (HPA). This case showed us that metabolic diseases may accompany a delay in the diagnosis of SCID and patients should be evaluated with a multidisciplinary approach.

## 1. Introduction

Severe combined immunodeficiency (SCID) is a rare group of diseases that are clinically more severe among inborn errors of immunity (IEI) and are usually diagnosed in childhood [1]. In recent years, with the widespread use of next generation sequencing (NGS), there has been an increase in the diagnosis of adult-onset SCID. In SCID characterized by life-threatening infections (bacterial, viral, or fungal), profound T cell deficiency may be accompanied by B cell and/or natural killer (NK) cell deficiencies [2, 3]. In other words, four subgroups have been defined in SCID based on immunophenotypes: T−B−NK−, T−B−NK+, T−B+NK−, and T−B+NK+. Stem cell transplantation, gene therapy, and enzyme replacement therapy are the treatment modalities that provide a cure for SCID [4].

Leaky SCID is characterized by partial T-cell deficiency and its diagnosis may be delayed until adulthood [2]. The atypical clinical presentation in this condition results from hypomorphic mutations or mutations that have not yet been identified. The criteria for the diagnosis of leaky SCID were defined by the primary immunodeficiency (PID) treatment consortium (PIDTC) and published in 2022. Atypical disease has been shown to be caused largely by variants detected in recombination activating 1 (RAG1), RAG2, adenosine deaminase (ADA), RNA component of mitochondrial RNA processing (RMRP) endoribonuclease, and DNA cross-link repair 1C (DCLRE1C) genes [5].

Inborn errors of metabolism (IEM) disorders are a heterogeneous group of diseases that occur due to deficiencies or functional losses of enzymes involved in various biological processes and affect various organs and systems [6]. For instance, since molecules involved in amino acid metabolism regulate the effector functions of immune cells, defects that disrupt amino acid metabolism can lead to various deficiencies or functional disorders in the immune system. The majority of IEM disorders are monogenic diseases and are transmitted to descendants by autosomal recessive inheritance. One of these diseases, hyperphenylalaninemia (HPA), is a hereditary amino acid metabolism disorder caused by the absence or insufficiency of the phenylalanine hydroxylase (PAH) enzyme. Increased phenylalanine and metabolites (phenylacetate, phenyllactate, and phenylpyruvate) resulting from disruption of phenylalanine metabolism accumulate in various organs, especially the brain, and cause tissue damage. HPA is roughly divided into three, depending on the severity of the clinical picture and the level of phenylalanine in the blood [7]. These are classic phenylketonuria (PKU; phenylalanine > 1200 μmol/L), nonclassic PKU (moderate PKU/mild HPA; 360 < phenylalanine < 1200 μmol/L), and untreated non-PKU HPA (120 < phenylalanine < 360 μmol/L). While PKU leads to intellectual disability, seizures, and behavioral disorders, some patients with HPA (who do not have untreated PKU) do not exhibit clinical features of PKU. There are no comprehensive clinical studies in the literature addressing the relationships between HPA and PIDs. However, there are few studies reporting decreased immunoglobulin levels and delayed-type hypersensitivity responses in patients with PKU [6, 8, 9].

In this article, we present a patient with atypical SCID due to DCLRE1C mutation accompanied by HPA. Our aim is to draw attention to the possible relationship between IEI and IEM and to discuss this with the support of literature.

## 2. Case Presentation

A 25-year-old male patient who applied to our clinic with complaints of cough, phlegm, shortness of breath, and palpitations stated that he gets sick frequently and has difficulty recovering. The patient's complaints have gradually increased over the last 5 months. The patient stated that he was often sick in his childhood, that is why he used a lot of antibiotics and even had his tonsils removed when he was 8 years old. The patient had severe pneumonia when he was in high school and approximately 500 cc of fluid was taken from both lungs. Bronchiectasis was detected in radiological examinations performed while he was hospitalized. The patient had been treated for nonallergic asthma during childhood and had polyps removed from his nose six times. No sensitivity was detected in allergy tests performed at this time. While the patient was under follow-up at the chest diseases clinic, a sweat test was performed for cystic fibrosis diagnosis and the result was found to be negative. In addition, the patient was questioned about aspirin sensitivity, and it was understood that he had no such complaint. The patient states that he was last hospitalized and treated for severe pneumonia 2 months ago. At this time, the levels of IgG, IgA, and IgM measured in the patient's blood were found to be within normal limits. After the patient was discharged, he was referred to our clinic for advanced immunological evaluation.

The patient is single and his parents are cousins (paternal aunt and maternal uncle). There were no diagnosed cases of immunodeficiency or metabolic disease in the patient's family or close relatives. However, his older sister died of an unknown cause during gallbladder stone surgery when she was 20 years old. He has an older brother who is alive and healthy. The patient does not have bad habits such as drinking, smoking, or drug abuse. On physical examination of the patient, there was congestion in the scleras, a hyperemic pharynx with a cobblestone appearance, and purulent discharge from the postnasal passage. Coarseness of breath sounds, prolonged expiration, widespread expiratory rhonchi, and fine crackles at the bases were auscultated in the lungs. Other physical examination findings were almost within normal limits.

All routine biochemical parameters measured in venous blood were almost within normal limits. According to complete blood count (CBC) with differential results at first admission, it was found that white blood cell: 7.3 × 10^3^/mm^3^ (4.5–11 × 10^3^), neutrophil: 3.3 × 10^3^/mm^3^ (2–7.8 × 10^3^), lymphocyte: 2.7 × 10^3^/mm^3^ (1–4 × 10^3^), monocyte: 0.4 × 10^3^/mm^3^ (0–1 × 10^3^), eosinophil: 0.7 × 10^3^/mm^3^ (0–1 × 10^3^), basophil: 0.1 × 10^3^/mm^3^ (0–0.2 × 10^3^), hemoglobin: 18.3 gr/dL (12.5–16), hematocrit: 53.5% (37–47), and platelet count: 211 × 10^3^/mm^3^ (150–400 × 10^3^). Acute phase reactants were higher than their reference values, such as C-reactive protein (CRP): 158 mg/L (0–5) and erythrocyte sedimentation rate (ESR): 44 mm/h (0–15 for male). Antinuclear antibody (ANA), antineutrophil cytoplasmic antibody (ANCA), and antithyroid antibody tests indicating autoimmunity were negative. In addition, thyroid-stimulating hormone (TSH) was at a normal level of 4.3 mU/L (0.3–4.9). Amino acid levels in plasma were measured using liquid chromatography mass spectrometer (LC-MS) method. While the phenylalanine level in plasma was found to be significantly high (272.4 µmol/L (30–80)), isoleucine and leucine levels were found to be slightly increased (112.7 µmol/L (36–107) and 202.4 µmol/L (68–183), respectively). Additionally, the phenylalanine/tyrosine ratio was observed to be slightly higher than the upper value of the reference limit (3.02 (0.2.5)).

Although serum levels of all major immunoglobulin isotypes and total IgE measurement were within normal limits at the time of diagnosis, some abnormal levels were detected in IgG subgroups. Accordingly, IgG: 16.7 g/L (6.5–16.3), IgA: 1.3 g/L (0.6–4.2), IgM: 1.4 g/L (0.3–2.9), total IgE < 10 IU/mL (<87), IgG1:11.1 g/L (4.05–10.11), IgG2:1.4 g/L (1.69–7.86), IgG3:0.3 g/L (0.11–0.85), and IgG4 < 0.02 g/L (0.03–2.01). While the patient's blood group was B Rh+, the anti-A antibody titer was lower than 1/8. The antipneumococcal antibody level of the patient, who was vaccinated 5 years ago with pneumococcal vaccine available on the market at the time, was 5.3 mU/mL (negative: <5, borderline: 5–7, and positive: >7). Since there was no protective level of antipneumococcal antibody, we administered a single dose of 13-valent conjugated pneumococcal vaccine to the patient.

In the lymphocyte subgroup analysis, while the percentages of CD3+ and CD8+ T cells (63.4% vs. (62–87); 25.3% vs. (15–46), respectively) and the CD4/CD8 ratio (0.93 vs. (0.8–3.9)) were within reference values, the percentage of CD4+T cell (23.6% vs. (32–64)) was found to be decreased compared to the lower limit of the reference values. In the same analyze, the percentage of CD19+ B cell was found to be deeply below the lower limit of normal values (0.9% vs. (6–26)), but the percentage of CD3−CD16+CD56+ NK cell was found to be above the upper limit of normal values (35.5% vs. (4–26)). In addition, the percentage of CD3+CD4−CD8− T cell was found to be higher than it should be (20.3% vs. (3.1–10.8)). Therefore, it was deemed necessary to analyze the distribution of T cell receptor (TCR) alpha–beta (TCR*αβ*) and TCR gamma-delta (TCR*γ*δ) bearing-T cells in peripheral blood.

Accordingly, while the percentage of TCR*γ*δ+ CD3+T cells was found to be high (21.2% vs. (0.6–12.3)), the percentages of TCR*αβ*+CD3+ T cells was found to be low (76.3% vs. (87.0–99.3)). Double negative T cells (DNTs: TCR*αβ*+CD3+CD4-CD8- T) were within normal limits (0.6 vs. (0.5–3.9)). Thus, it was understood that the high percentage of CD3+CD4−CD8− T cells detected in the patient's lymphocyte subgroup analysis was due to the high percentage of TCR*γ*δ+CD3+CD4−CD8− T cells. Naïve and memory T cells were also analyzed and, respectively, found as 1% for CD4+CD45RA+ T cells (8.2–73.3), 88.1% for CD4+CD45RO+ T cells (28.2–86.6), 22.3% for CD8+CD45RA+ T cells (19.3–86.2), and 39.5% for CD8+CD45RO+ T cells (11.5–72.5). It was also observed that the percentage of recent thymic emigrants (RTEs: CD4+CD45RA+CD31+) was below the lower limit of normal values (0.2% (10–56.7)). We defined the immunological composition of the patient as T−B−NK+ leaky SCID phenotype.

Computed tomography (CT) examination of the thorax was performed without the use of radiocontrast agents. In various lobes and segments of both lungs: tree-in-bud appearance compatible with bronchiolitis, areas of tubular bronchiectasis, peribronchial thickening, and areas of pneumonic infiltration were observed. In addition, there was a small amount of effusion in the pericardial sac (<10 mm). In the CT examination of the facial bones and sinuses: septum deviation, hypertrophy in the nasal turbinates, soft tissue appearances (polyposis) in the nasal passage, obliteration in bilateral osteomeatal complexes, and air loss in the paranasal sinuses (pansinusitis) were observed. In the entire abdomen examination by ultrasonography: parenchymal echogenicity and contours of the liver and spleen appeared normal and no solid or cystic lesion was detected. However, the size of the liver increased and the vertical length of the right lobe was measured as 168 mm. No pathological image was detected in other solid and hollow organs in the abdomen.

The patient's respiratory function tests were in a restrictive pattern (*z* scores: forced expiratory volume (FEV1)/forced vital capacity (FVC) > −1.64; FVC < −1.64), according to Global Lung Function Initiative 2012 reference values [10]. While FVC value was mildly affected (*z* scores: between −1.65 and −2.5), FEV1, maximal mid-expiratory flow (MMEF) 75–25, maximal expiratory flow (MEF) 50, and peak expiratory flow (PEF) values were moderately affected (*z* scores: between −2.5 and −4).

In bronchoscopy, the tracheal lumen was open and dense purulent secretion from bilateral main bronchi was observed. Bronchoalveolar lavage (BAL) and biopsy specimens were received for microbiological, cytological, and histopathological examinations. There was no growth of pathogenic bacteria or fungi in the cultures prepared from BAL samples. In cytological and histopathological investigations of these specimens, abundant neutrophils were observed along with bronchial epithelial cells without any signs of atypia and sparse alveolar macrophages.

Whole exome sequencing (WES) was used for molecular diagnosis. Exome enrichment was performed using Twist Comprehensive Human Exome kit according to manufacturer's instructions. Prepared library was sequenced on MGI-T7 at 80–100x on-target depth with 150 bp paired-end sequencing at Intergen Genetic Diagnostic Centre Ankara, Türkiye. Variants were classified by the American College of Medical Genetics and Genomics (ACMG) criteria. Potential candidates were confirmed using targeted sequencing on Illumina MiSeq platform. Confirmed candidate variants were also tested with the same methods amongst the members of the family. Accordingly, in WES study, three different variants, two homozygous and one heterozygous, were detected in our patient. These were DCLRE1C; NM_001033855.3 c.207_209del (p.Leu70del) – rs753202682- homozygous and TNF Receptor Superfamily Member 13B (TNFRSF13B); NM_012452.3 c.260T >A (p.Ile87Asn)–rs72553877 heterozygous variant in genes related to the immune system; and PAH: NM_000277.3 c.688G >A (p.Val230Ile)–rs62516152 homozygous variant in genes related to amino acid metabolism.

Until the genetic test (WES) results were received, the patient was evaluated as having IgG subgroup deficiency according to European Society for Immunodeficiencies (ESID) diagnostic criteria [11]. After WES analysis, clinical diagnosis changed with DCLRE1C/Artemis deficiency and HPA. We initially started intravenous immunoglobulin (IVIG) treatment at 600 mg/kg every 3 weeks after receiving off-label use approval from the Ministry of Health. In addition, prophylactic antibacterial and antifungal drugs were used. Considering the recommendations in the guideline published in 2017, no special medical diet was planned for our patient to keep the blood phenylalanine level within normal limits [12]. On the other hand, the treatments initiated by the chest diseases clinic before the patient applied to our clinic were continued at the same doses (inhaled corticosteroid/long-acting beta agonist and leukotriene antagonist). In the follow-up examination performed 6 months later, it was observed that the patient's respiratory complaints did not regress, and the lung findings and high eosinophil count in the blood continued. Therefore, it was decided to administer mepolizumab treatment subcutaneously at 100 mg per month. A dramatic regression was observed in the patient's complaints, clinical findings and eosinophil count in the blood at the first month of biological treatment.

## 3. Discussion

DCLRE1C is the gene encoding the Artemis protein, which is involved in the repair of double-stranded DNA breaks induced by RAG1 and RAG2 during T cell and B cell developments [5, 13]. The mutations in this gene cause radiosensitive form of SCID in the patients. In recent years, it was shown that hypomorphic mutations in DCLRE1C gene can cause a late-onset immunodeficiency that is milder phenotype than typical SCID such as leaky SCID, Omenn syndrome, hyper IgM syndrome, and inflammatory bowel disease. In hypomorphic mutations, the expression level or function of the wild-type gene product is partially sufficient. Therefore, in mutations such as this, the clinical course of the disease is milder and disease symptoms appear at later ages [14].

The variant (c.207_209del) we detected in our patient causes deletion of 1 amino acid of the DCLRE1C protein (p.Leu70del), but does not cause a change in the integrity of the reading frame (ClinVar). The frequency of this variant (rs753202682) in the Exome Aggregation Consortium (ExAC) database is 0.02%. It is not yet known how this variant, which has been shown to increase sensitivity to ionizing radiation in fibroblasts, affects the structure and function of the DCLRE1C protein [15]. Since the contribution of this variant to the disease pathogenesis has not yet been fully elucidated, it was classified as a variant of uncertain significance (VUS).

Although we did not show the level of Artemis expression, the fact that our patient was diagnosed with PID in adulthood and that the disease is fatal in patients with DCLRE1C mutation who did not undergo stem cell transplantation in infancy supports that the mutation carried by our patient is hypomorphic. From another perspective, this variant can be classified as likely pathogenic, as the patient's clinical presentation supports the diagnosis of PID. To reveal which of these possibilities is valid, functional studies should be performed on our patient and on other patients carrying our patient's variant and their families.

The TNFRSF13B gene encodes the transmembrane activator and calcium modulator and cyclophilin ligand interactor (TACI) molecule, which belongs to the TNF receptor superfamily on the B lymphocyte surface and interacts with a proliferation-inducing ligand (APRIL) and B-cell activating factor (BAFF) [13]. The TACI molecule plays a role in the differentiation of B cells, immunoglobulin production and isotype switching, as well as the removal of autoreactive B cells. Heterozygous and homozygous loss-of-function mutations identified in this gene have been shown to be associated with CVID.

It has been reported that the variant (c.260T >A), we detected in the TNFRSF13B gene in our patient, was inherited in heterozygous or compound heterozygous state (ClinVar) in patients with CVID and IgA deficiency [16]. The frequency of this variant (rs72553877) in the Genome Aggregation Database (gnomAD) is 0.068% in the non-Finnish European population. Functional studies have shown that the change in protein structure caused by this variant (p.Ile87Asn) reduces nuclear factor kappa B (NF*κ*B) signaling in cells [17]. Therefore, this variant was classified as likely pathogenic. However, there are also laboratories that accept this variant as VUS. It is well known that heterozygous variants are not always innocent in some genetic disorders. Therefore, we classified this variant as pathogenic and wanted to include it in our report to discuss its possible impact on clinical and immunological features. However, the detection of two different mutations thought to cause PID in the same patient has made it difficult to discuss this issue on a single patient basis.

The immunological findings we detected in our patient reflect the possible common effects of the variants carried by the patient. The low number of Naive T cells in our patient's peripheral blood is due to the severe decrease in RTEs, which indicates that thymus functions are seriously reduced. In addition, our patient's B cells have decreased significantly in the peripheral blood. Accordingly, deficiencies were detected in IgG2 and IgG4 subgroups. If we were to define this composition immunologically, we can say that our patient partially fits the T−B−NK+ SCID immune phenotype [5].

Phenylalanine, an essential amino acid, has been shown to have effects on various immune functions in experimental and clinical studies [18]. PAH: NM_000277.3 c.688G >A (p.Val230Ile)-rs62516152 homozygous variant found in our patient was classified as benign, likely benign, VUS, likely pathogenic, and pathogenic in ClinVar database. As this variant is not causing classical PKU but HPA, this confusion is not surprising. Despite our case has increased blood phenylalanine levels and phenylalanine/tyrosine ratio without a clinical feature of PKU (untreated non-PKU HPA), this variant was classified as pathogenic for HPA. Although studies showing the relationship between HPA and PID are lacking in the literature, the results of two old-dated studies on this subject are noteworthy. In one of these, Passwell et al. [8] found that serum immunoglobulins were low in patients with PKU, but delayed-type skin sensitization to purified protein derivative (PPD) was normal. In the other study, Karagoz et al. [9] found that delayed-type skin sensitization to PPD was reduced and serum IgG and IgM levels were low in patients with PKU.

In recent years, the developments in the fields of molecular biology and genetics at an unprecedented pace and the current use of high-resolution technologies have brought to the fore complex diseases in which more than one genetic defect is detected in the same patient [19]. The coexistence of more than one genetic defect in the same patient may be due to causal relationships between these defects or may be due to chance or selection bias. Our patient has two different genetic disorders (DCLRE1C/Artemis deficiency and HPA) and three likely pathogenic/pathogenic variants. In the literature, a 10-month-old male patient diagnosed with Artemis deficiency and 3 M syndrome has been reported from Türkiye [20]. In the patient who was born from consanguineous marriage and had signs and symptoms of neuromotor developmental delay and immune deficiency: mild lymphopenia, hypogammaglobulinemia, decreased number of CD3+ T cells (980 cells/mm^3^), and CD19+ B cells (35 cells/mm^3^) were detected. In the WES analysis of the patient who was finally evaluated as leaking T−B−NK+ SCID: homozygous pathogenic variants in DCLRE1C gene (c.194C>T; p.T65I (NM_001033855)) and obscurin like cytoskeletal adaptor 1 (OBSL1) gene (c.3922C>T; p.R1308X (NM_001173431)) were shown. The authors state that this case was lost due to sepsis.

In conclusion, this case showed that leaky SCID may be accompanied by different immunological and metabolic defects. The comprehensive clinical and experimental studies revealing the relationships between clinical, genetic, immunological, and biochemical parameters are needed to reveal whether there is a causal relationship between the genetic variants affecting both immunity and metabolism. In addition, the management of the disease in patients with multiple genetic defects is as difficult as understanding the mechanism of the disease and to diagnose it. These issues can only be overcome with a multidisciplinary approach.

## Data Availability

The data that support the findings of this study are available from the corresponding author upon reasonable request.
